# Single-trial classification of evoked responses to auditory tones using OPM- and SQUID-MEG

**DOI:** 10.1088/1741-2552/acfcd9

**Published:** 2023-10-06

**Authors:** Joonas Iivanainen, Tony R Carter, Michael C S Trumbo, Jim McKay, Samu Taulu, Jun Wang, Julia M Stephen, Peter D D Schwindt, Amir Borna

**Affiliations:** 1 Sandia National Laboratories, Albuquerque, NM 87185, United States of America; 2 Candoo Systems Inc, Port Coquitlam, BC, Canada; 3 University of Washington, Seattle, WA, United States of America; 4 Department of Speech, Language, and Hearing Sciences, The University of Texas at Austin, Austin, TX, United States of America; 5 Department of Neurology, The University of Texas at Austin, Austin, TX, United States of America; 6 The Mind Research Network a Division of Lovelace Biomedical Research Institute, Albuquerque, NM 87106, United States of America

**Keywords:** magnetoencephalography, optically pumped magnetometer, classification, decoding, brain–computer interface, auditory cortex

## Abstract

**Objective.:**

Optically pumped magnetometers (OPMs) are emerging as a near-room-temperature alternative to superconducting quantum interference devices (SQUIDs) for magnetoencephalography (MEG). In contrast to SQUIDs, OPMs can be placed in a close proximity to subject’s scalp potentially increasing the signal-to-noise ratio and spatial resolution of MEG. However, experimental demonstrations of these suggested benefits are still scarce. Here, to compare a 24-channel OPM-MEG system to a commercial whole-head SQUID system in a data-driven way, we quantified their performance in classifying single-trial evoked responses.

**Approach.:**

We measured evoked responses to three auditory tones in six participants using both OPM- and SQUID-MEG systems. We performed pairwise temporal classification of the single-trial responses with linear discriminant analysis as well as multiclass classification with both EEGNet convolutional neural network and xDAWN decoding.

**Main results.:**

OPMs provided higher classification accuracies than SQUIDs having a similar coverage of the left hemisphere of the participant. However, the SQUID sensors covering the whole helmet had classification scores larger than those of OPMs for two of the tone pairs, demonstrating the benefits of a whole-head measurement.

**Significance.:**

The results demonstrate that the current OPM-MEG system provides high-quality data about the brain with room for improvement for high bandwidth non-invasive brain–computer interfacing.

## 1. Introduction

Magnetoencephalography (MEG) is a noninvasive neuroimaging technique in which the magnetic fields of the human brain are detected outside the head (Hämäläinen et al 1993). Due to the weakness of the neuromagnetic fields (about 10 fT to 1 pT), sophisticated magnetometers with sensitivities around a few fT/rt-Hz are needed. Historically, superconducting quantum interference devices (SQUIDs) were the only sensor sensitive enough to register the neuromagnetic fields (e.g. Koshev et al 2021). Current commercial, clinical-grade MEG systems utilize hundreds of SQUID sensors around the head for spatial sampling of the field with sensor sensitivity of about 2 fT/rt-Hz.

The use of cryogenics necessitated by liquid helium cooling of the SQUIDs imposes several drawbacks for the SQUID-based MEG systems. First, the thermal insulation needed between the participant’s head and the superconductive sensors introduces a gap of at least 2 cm between the scalp and sensors limiting the measured signal amplitudes as well as the spatial resolution (Iivanainen et al 2017). Second, the one-size-fits-all SQUID sensor helmet cannot be reconfigured to individual head shape or size further limiting the spatial resolution especially in children and women (e.g. Hill et al 2019, Koshev et al 2021, Marhl et al 2021). Third, the heavy SQUID-MEG systems do not allow scanning of moving or ambulating participants beyond the limits of the rigid helmet (Boto et al 2018).

Recently, optically pumped magnetometers (OPMs; Budker and Romalis 2007) have emerged as a near-room-temperature alternative to SQUIDs. In contrast to SQUIDs, OPMs enable wearable sensor arrays detecting the field within millimeters from the scalp increasing the usability and spatial resolution of MEG (Iivanainen et al 2017, Boto et al 2018). To date, numerous OPM sensors with various designs have been proposed for detection of MEG with sensitivities around 10 fT/rt-Hz and bandwidths of 100–200 Hz (e.g. Shah and Wakai 2013, Colombo et al 2016, Knappe et al 2023). The individual OPM sensors have been successfully assembled into MEG sensor arrays having partial (Borna et al 2017, Tierney et al 2018, Iivanainen et al 2019, 2020, An et al 2022a, An et al 2022b, Gutteling et al 2023, Hillebrand et al 2023) or full (Hill et al 2020, Pratt et al 2021, Alem et al 2023) coverage of the head.

Despite the plethora of simulations (Boto et al 2016, Iivanainen et al 2017, 2021, Tierney et al 2020, Beltrachini et al 2021, Bezsudnova et al 2022, Marhl et al 2022, Nugent et al 2022, Zahran et al 2022) and experimental studies comparing OPM- and SQUID-based MEG, it is still not fully and comprehensively understood how these measurement sensors compare. On the one hand, OPMs are closer to the brain, thus picking up higher signal amplitudes and potentially demonstrating higher spatial resolution than SQUIDs. On the other hand, SQUIDs have an advantage over OPMs in terms of sensor sensitivity, bandwidth, and coverage. In addition, SQUID-MEG systems are mature and well understood while OPMs may suffer from systematic effects (Borna et al 2022).

Here, to compare our OPM-MEG system (Borna et al 2017) to a commercial SQUID-MEG system in a data-driven way, we quantified their performance in classifying single-trial evoked responses to auditory stimulation. Similar approach has been previously used to compare an on-scalp MEG system based on high-*T*_c_ SQUIDs to a commercial low-*T*_c_ SQUID system in classifying responses from the somatosensory cortex (Andersen et al 2020). We recorded auditory evoked fields (AEFs) to three sinusoidal tones from six participants and used machine-learning techniques to classify the measured responses to the three tone classes.

The single-trial classification performance quantifies the signal-to-noise ratio (SNR) and spatial resolution of the sensor arrays as well as the data quality (due to complex factors such as participant comfort, head motions and external interference) yielded by the system. Additionally, the classification accuracy serves as an indicator of the system’s performance in brain–computer interfacing where single-trial responses need to be decoded real time to control an external device. Single-trial evoked responses are also of interest in naturalistic stimulus paradigms (Hari et al 2015) where the responses need to be correlated with the environment and participant’s current state, as well as in estimating how the brain background activity influences the evoked responses (Mazaheri and Jensen 2010).

## 2. Materials and methods

In this section, we outline the methods for data acquisition, preprocessing, and analysis. We analyzed the evoked responses to the auditory tones for each participant. For classification analysis, we use three different approaches: linear discriminant analysis (LDA) combined with principal component analysis (PCA), a convolutional neural network (EEGNet; Lawhern et al 2018), and xDAWN spatial filtering (Rivet et al 2009) combined with Riemannian geometry (Barachant et al 2011) and logistic regression.

### 2.1. Participants

Six healthy adult volunteer participants (three males and three females, 28–46 years of age) with no known history of neurological or psychiatric disorders participated in the study. The protocols of the MEG experiments were approved by the Human Studies Board of Sandia National Laboratories and the Chesapeake Institutional Review Board. Prior to experiments, informed written consent was obtained by the project’s primary investigator from all the participants.

### 2.2. Experimental paradigm and stimuli

To elicit AEFs, auditory tones were presented to the participant’s ears binaurally using non-magnetic 50 Ω Insert earphones (Etymotic Research, Inc., US). We used sinusoidal tone pulses at three different frequencies (0.5, 1, and 4 kHz). The pulse durations of the tones were set to 50 ms and the interstimulus interval was 650 ms with a random jitter of 100 ms. For each tone, approximately 500 stimuli were presented. In both SQUID- and OPM-MEG systems, the stimuli were presented via the stimulus delivery program Presentation (Neurobehavioral Systems, US).

AEFs are typically grouped according to their latency after the stimulus onset (Hari and Puce 2023). The earliest responses are from the brainstem and they occur within the first 8–10 ms. They are followed by middle-latency auditory-evoked fields within 12–50 ms. The first robust cortical responses is in this category and can be recorded at 18–19 ms (here denoted as M20). M50 is another robust response peaking at about 50 ms also included in this category. Long-latency auditory-evoked fields occur later from about 50 to 250 ms. The most prominent auditory response occurs at around 100 ms (M100) which is usually followed by a deflection of opposite polarity (M200).

### 2.3. MEG acquisition

#### 2.3.1. OPM

**Figure 1.**
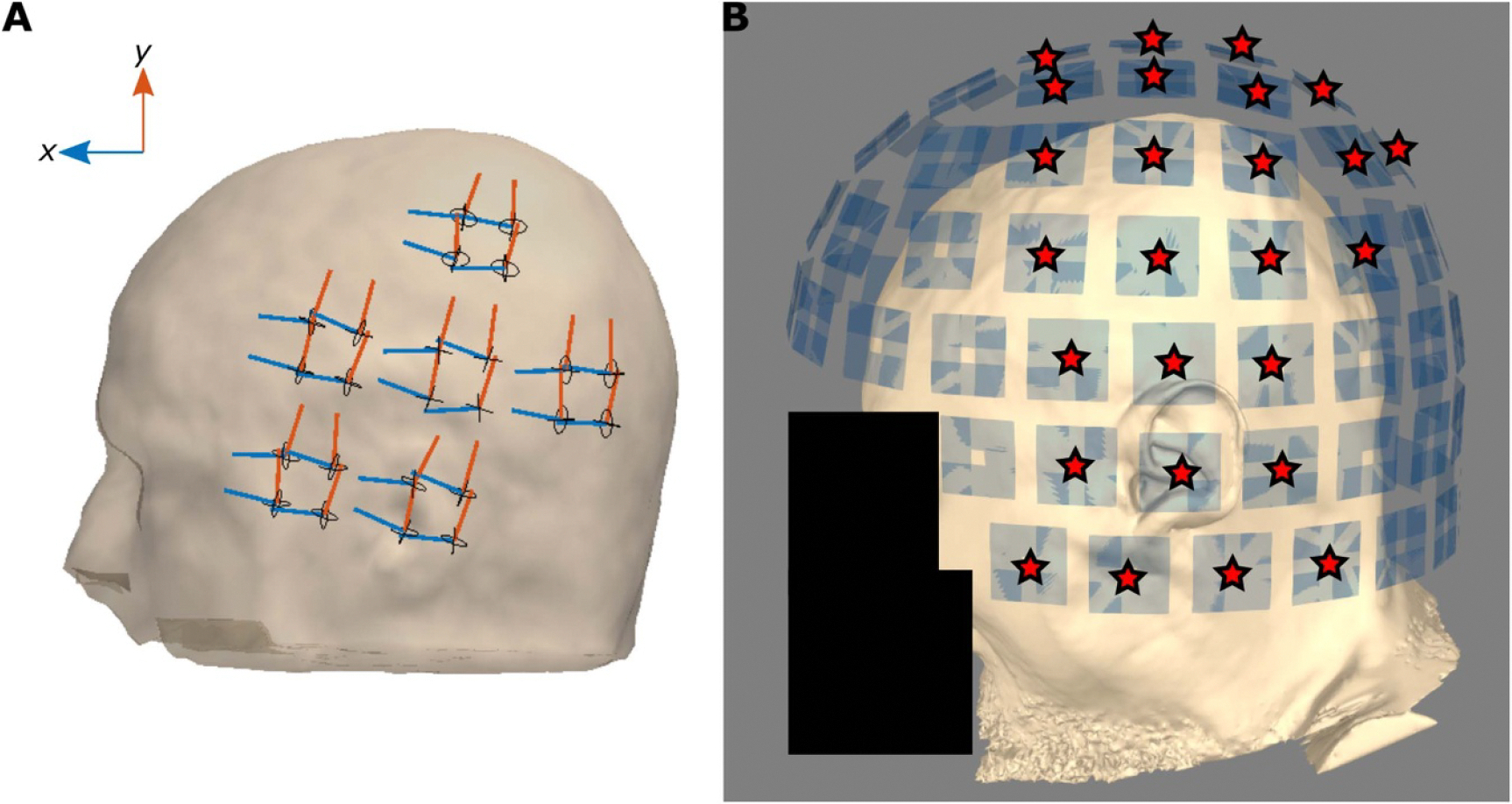
Illustrations of OPM and SQUID sensor arrays used to measure AEFs. (A): The 24-channel OPM array used to measure AEFs from participant’s left auditory cortex. The 24 channels measure either one of the tangential field components with respect to the participant’s head surface (denoted as xOPM and yOPM). The sensitive directions of xOPM and yOPM are indicated in the figure. Black circles and the colored arrows show the OPM sensor locations and orientations, respectively. (B): SQUID sensor array with respect to an example head. Blue rectangles illustrate the SQUID sensors. Left temporal parietal SQUID sensors are marked with stars (mSQUID-LPT: 26 magnetometers; gSQUID-LPT: 52 planar gradiometers). The head geometry and the MEG coregistration to the magnetic resonance images is from the example data of MNE software (Gramfort et al 2014).

We recorded the participants’ AEFs using our OPM-MEG system at Sandia National Laboratories (Borna et al 2017, 2020). Briefly, the OPM-MEG system consists of six 4-channel OPMs in a sensor helmet covering the participant’s left hemisphere, a person-sized cylindrical magnetic shield with embedded shield coils for field cancellation as well as electronics for data acquisition and stimulus presentation. The OPM sensors have a magnetic (gradiometrically inferred) sensitivity of ~10 fT/rt-Hz (5 fT/rt-Hz) and bandwidth of around 90 Hz (Colombo et al 2016). Due to the design of the OPM, the OPM channels measure one field component tangential to the participant’s scalp surface; we measured two approximately orthogonal tangential components in sequential measurements. The total number of channels in the array is 24, which measure either one of the tangential components (denoted as xOPM and yOPM, respectively). The participant was positioned in the OPM helmet so that the OPMs covered the region of the scalp where we estimated the AEFs to be the strongest. Figure 1(A) gives an illustration of the OPM array with respect to a participant’s brain surface.

Prior to the measurement, the OPMs were heated to their operating temperature of about 150 °C, the magnetic field was zeroed in the sensor array, and the OPMs were calibrated using the shield coils (Borna et al 2017). The OPM photodiode outputs were sampled at 100 kS s^−1^ and were processed with software lock-in amplifiers that output the OPM magnetometer signals at 1 kS s^−1^ (Borna et al 2017).

#### 2.3.2. SQUID

The same participants were also measured with a commercial cryogenic MEGIN-Neuromag SQUID system (MEGIN Oy, Espoo, Finland) located inside a three-layer magnetically shielded room (AK3b, Vacuumschmelze GmbH & Co., Hanau, Germany) at the Mind Research Network (Albuquerque, NM, USA). The SQUID-MEG system consists of 306 channels configured on 102 sensor elements in the sensor helmet (each element has a sensor triplet measuring the normal component of the magnetic field and its tangential planar gradients). We denote the 102 magnetometers of the helmet as mSQUID-ALL and the 204 planar gradiometers as gSQUID-ALL. SQUID-MEG was acquired at 1 kS s^−1^ with a low-pass filter at 330 Hz.

## 2.4. Data analysis

### 2.4.1. Data preprocessing

The SQUID data were preprocessed using the MaxFilter software (MEGIN Oy, Espoo, Finland) to suppress external magnetic interference (Taulu and Simola 2006). The subsequent OPM and SQUID data preprocessing was performed using the MNE Python software (Gramfort et al 2014).

For the evoked and LDA analysis, the OPM and SQUID data were band-pass filtered at 1–90 Hz (for one participant low-pass cutoff at 70 Hz was used due to noise peaks in the OPM data above 70 Hz). The data for classification with EEGNet and xDAWN spatial filtering were band-pass filtered at 1–43 Hz and downsampled to 128 Hz. All the data were also notch filtered at the line frequency (60 Hz) and its harmonics up to 240 Hz.

Independent component analysis (ICA; Hyvärinen and Oja 2000) was applied to remove interference components from both the OPM and SQUID data. ICA components were computed using the filtered data, the components were visually inspected, and those deemed to represent interference were removed.

Time-locked individual epochs were extracted between −0.2 s and 0.5 s relative to the stimulus trigger signal at *t* = 0 s. The epochs were time-shifted to correct for the latency between the stimulus trigger, stimulus presentation and the processed sensor output. For OPMs and SQUIDs, the latencies between the trigger and the processed sensor outputs were measured to be about 5 ms and 43 ms, respectively. The individual epochs were baseline corrected by subtracting the average signal before stimulus onset (−0.2–0 s). Noisy epochs were removed by a threshold-based rejection: the epochs in which the peak-to-peak amplitude of any of the channels exceeded the chosen threshold were discarded from the analysis. The peak-to-peak rejection thresholds were determined for each participant and sensor type separately.

### 2.4.2. Evoked responses

For visualization and temporal/amplitude analysis of the evoked responses, the epochs obtained as described in the previous section were averaged over individual trials. Please note that in the evoked analysis the numbers of trials in the averages between different sensor types and subjects were different due to different recording times and rejection thresholds; for the classification analysis we balanced the trial numbers across the sensors (see section 2.4.3). To compare the SNRs of the single-trial evoked responses between the systems, we estimated the single-trial SNRs by dividing the peak-to-peak amplitude of the average response by the standard deviation of the pre-stimulus baseline data across all trials.

We also compared the field patterns of the evoked responses measured with OPMs and SQUIDs. To visualize the 2D tangential field pattern measured with OPMs, at each channel location we multiplied the calibrated sensitive axis of the OPM channel with the measured signal and added the resulting vectors from xOPM and yOPM. We color coded the field-pattern plots using the magnitude (norm) of the 2D field.

### 2.4.3. Pairwise classification with LDA

For the classification analyses, we investigated two different SQUID-sensor layouts. For the first layout, we included all the SQUID channels in the helmet (separate arrays for magnetometers and gradiometers: mSQUID-ALL and gSQUID-ALL, respectively). For the second layout, we restricted the SQUID channels so that they had a similar coverage of the participants’ heads to match that of the OPM-MEG system. For this purpose, we considered the SQUID channels located around the left side of the helmet covering approximately the left temporal and parietal cortices of the participant (mSQUID-LPT: 26 channels; gSQUID-LPT: 52 channels; figure 1(B)). It should be noted that ICA and epoch rejection were applied using data from mSQUID-ALL and gSQUID-ALL after which the channel selection was performed to get mSQUID-LPT and gSQUID-LPT data.

We performed pairwise classification of the single-trial responses to the three tones using LDA. LDA classifier fits a linear decision boundary between the classes by assuming that the class data are Gaussian distributed with equal covariance matrix for each class.

Prior to the analysis, we applied the following procedure for each participant’s epochs (section 2.4.1.) to use the same amount of data for classification with each sensor array. First, for each sensor array (xOPM, yOPM, mSQUID-ALL, etc), the number of epochs was balanced across the tones (stimulus classes) by discarding epochs until the classes had the same number of epochs. Second, the number of epochs across the sensor arrays were also balanced so that all the sensor types had the same number of epochs as the sensor type with the smallest number of epochs. The epochs were dropped according to their temporal occurrence, those corresponding to earlier time points were included. In this way, the number of epochs is different for each participant, but the different sensor arrays corresponding to a single participant had the same amount of data.

Next, the single-trial epochs were downsampled to 500 Hz to reduce the computational burden and were cropped to −0.1–0.4 s around the stimulus onset. PCA was applied to the resulting epochs for feature selection and dimensionality reduction; PCA components explaining 99% variance were included in the classification. Before and after the application of PCA, the mean was removed from the signals, and they were scaled to unit variance.

We trained an LDA classifier (implemented in Scikit-learn Python module; Pedregosa et al 2011) for each time point and tone pair. Default parameters were used for the classifier: singular-value decomposition solver, no shrinkage, no priors and default tolerance (1 × 10^−4^). The generalization of the classifier was assessed using a stratified ten-fold cross-validation with a 9:1 training-to-test ratio. The average classification accuracy over the ten cross-validation folds was calculated as the final accuracy. The sensor-level field maps of the discriminant neural sources were extracted from the classifier (Haufe et al 2014).

### 2.4.4. Multiclass classification with EEGNet and xDAWN spatial filtering

We used both EEGNet (Lawhern et al 2018) and a combination of xDAWN spatial filtering (Rivet et al 2009), Riemannian geometry (Barachant et al 2011), and logistic regression to perform multiclass classification of the responses to the three tones. EEGNet is a compact convolutional neural network that uses temporal and depthwise convolutions to learn frequency filters and frequency-specific spatial filters from the data, respectively. Separable and pointwise convolutions are used to summarize the feature maps and mix them optimally. The model has a few parameters: *F*_1_ and *F*_2_ control the number of temporal and pointwise filters to learn, respectively, *D* controls the number of spatial filters to learn within each temporal convolution, kernel length is the length of the temporal convolution, and dropout rate controls the probability of randomly dropping a unit from the network to prevent overfitting.

The epochs downsampled to 128 Hz were used, and for each participant the stimulus classes as well as the epoch counts of different sensor types were balanced as described in section 2.4.3. The epochs were cropped to 0–0.4 s around the stimulus onset.

The EEGNet implementation provided in GitHub (https://github.com/vlawhern/arl-eegmodels) was used with a configuration (*F*_1_ = 8, *D* = 2, *F*_2_ = 16), a kernel length of 64 samples and a dropout rate of 0.5. A four-fold stratified cross-validation was performed with a training:validation:test split ratio of 2:1:1; we report average classification accuracies over the four folds. The EEGNet model is trained in Tensorflow (Abadi et al 2016) with Adam optimizer minimizing the categorical cross-entropy loss function. Batch size of 16 was used and 500 training epochs were performed with a validation stopping (Lawhern et al 2018).

We used pyRiemann Python package (Barachant et al 2022) to estimate xDAWN covariance matrices and to project them to their tangent space. After projection, the features were scaled using MinMaxScaler (Scikit-learn) and logistic regression with *l*_2_-penalty was applied for classification (LogisticRegression function in Scikit-learn; multinomial loss; lbfgs solver). As with EEGNet, four-fold stratified cross-validation was performed but with a training:test split ratio of 3:1. Number of xDAWN spatial filters estimated from the training data ranged from one to seven. For every sensor type and participant, we report the classification accuracy for the number of spatial filters that gave the best average accuracy over the cross-validation folds.

### 2.4.5. Statistical analysis

Participant-level statistical significance of the LDA classification accuracy was assessed with a permutation test where the class labels where randomly permuted 300 times. The *p*-values obtained from the permutation test were false discovery rate (FDR) corrected for multiple comparisons with *q* = 0.05 (Benjamini and Hochberg 1995).

Group-level statistical significance was assessed using the Wilcoxon signed-rank test. To evaluate whether the classifier performed above chance level, single-sided tests were used; for comparison between the sensor arrays two-sided tests were applied. The temporal group-level LDA classifier *p*-values were FDR-corrected for multiple comparisons with *q* = 0.05 (Benjamini and Hochberg 1995).

## 3. Results

In the following, we will first present the results for a single participant and then summarize them over all the participants. The results for all individual participants can be found in the supplementary material.

After the epoch-equalizing procedure across the arrays, the total number of class-balanced epochs per tone ranged from 247 to 482 across the six participants (median: 356.5). The median number of ICA components removed across the participants was 5, 5.5, 2.5 and 2 for xOPM, yOPM, mSQUID-ALL and gSQUID-ALL, respectively. The corresponding ranges of removed ICA components were 4–5, 4–6, 2–4 and 1–3, respectively. The removed ICA components from the SQUID data mostly corresponded to the participant’s heartbeat and eye blinks, while OPM ICA components showed more variability including interference due to shield vibrations (see Borna et al 2020). The threshold-based rejection of epochs, on average over the subjects, led to rejection of 2.5% (0.6%–4.6%), 0.8% (0.4%–1.3%), 0.8% (0.4%–2.3%) and 0.7% (0.1%–1.7%) of epochs in xOPM, yOPM, mSQUID-ALL and gSQUID-ALL, respectively.

### 3.1. Evoked responses

**Figure 2.**
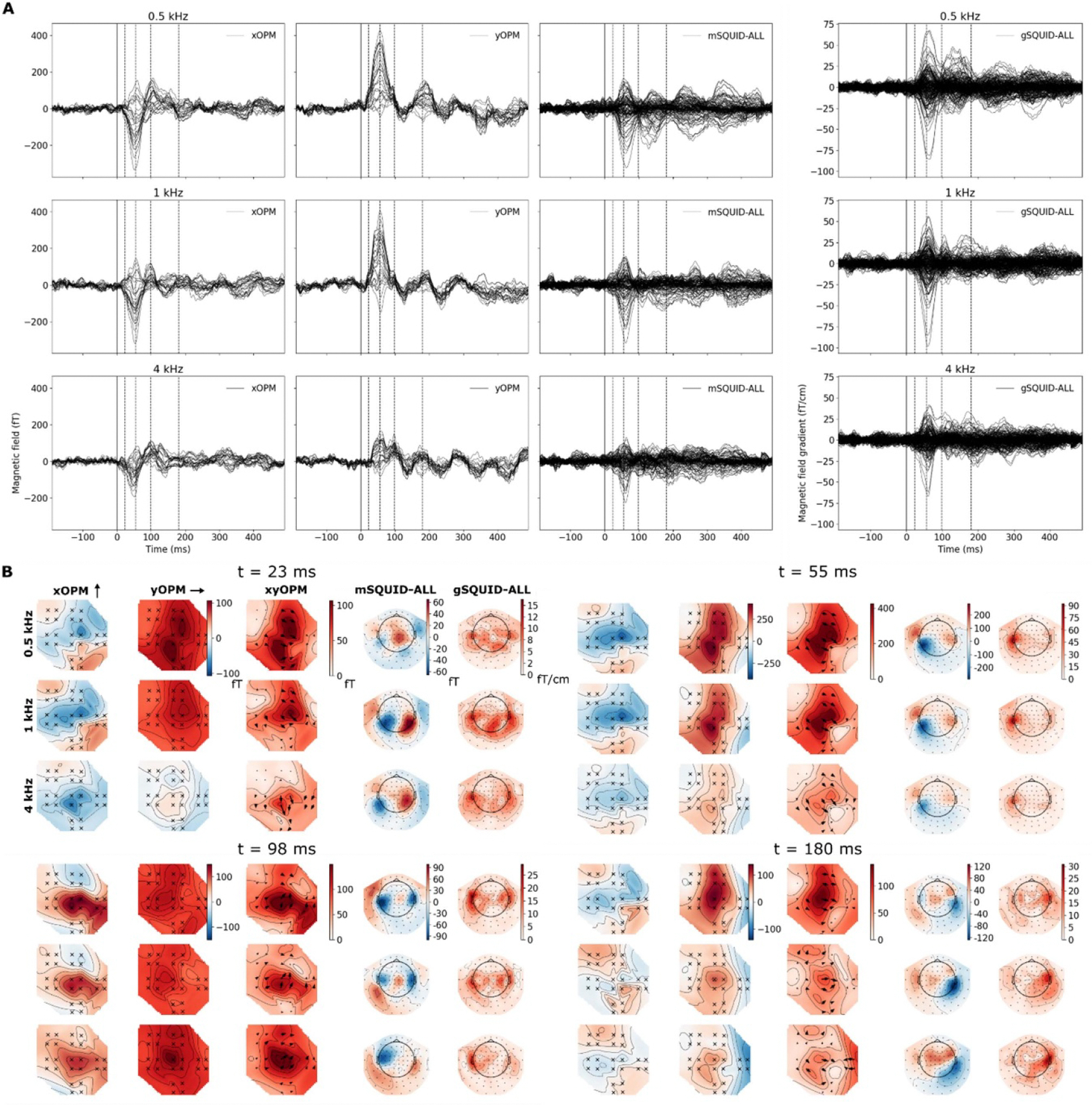
Single participant averaged evoked responses to 0.5, 1 and 4 kHz tones measured using OPM and SQUID sensor arrays (xOPM, yOPM: 24 OPMs measuring a tangential field component indicated in the figure with an arrow; mSQUID-ALL: 102 SQUID magnetometers; gSQUID-ALL 204 planar SQUID gradiometers). (A): Butterfly plots of the evoked responses as a function of time. Columns correspond to sensor arrays while rows correspond to tones. (B): Field plots of the evoked responses at four time instances (23, 55, 98 and 180 ms; dashed vertical lines in (A)). The field maps for SQUID arrays are scaled individually to their absolute maximum value; for OPMs each row at the same time instant has the same scale. The two tangential OPM components are combined in field maps denoted with ‘xyOPM’: the color indicates the norm of the tangential field vector while the arrow shows its direction. Positive/negative values are shown in red/blue, respectively.

Figure 2(A) shows the averaged evoked responses to the three tones for a single participant. The earliest OPM response to the tone is around 23 ms (M20), while the maximum response occurs around 50–60 ms (M50). Similarly, the maximum SQUID response is around 60 ms (M50). The participant also shows response around 100 ms (M100) which is well visible in the xOPM data. The OPM peak responses are slightly larger or comparable to those of mSQUID-ALL: for example, for 0.5 kHz tone, the maximum responses are 330, 430 and 320 fT for xOPM, yOPM and mSQUID-ALL, respectively.

Figure 2(B) provides examples of the field patterns of the evoked responses. The OPMs show similar responses to the three tones at 55 ms consistent with a dipolar field pattern with extrapolated radial component maximal in the corners of the OPM array and tangential maximum at the center of the array (figure 2(B): xyOPM column). The SQUID M50 responses show a similar dipolar field pattern at the left hemisphere of the subject. OPM and SQUID M100 responses show field patterns that have rotated approximately 90° from the M50 response on the sensor surface.

**Figure 3.**
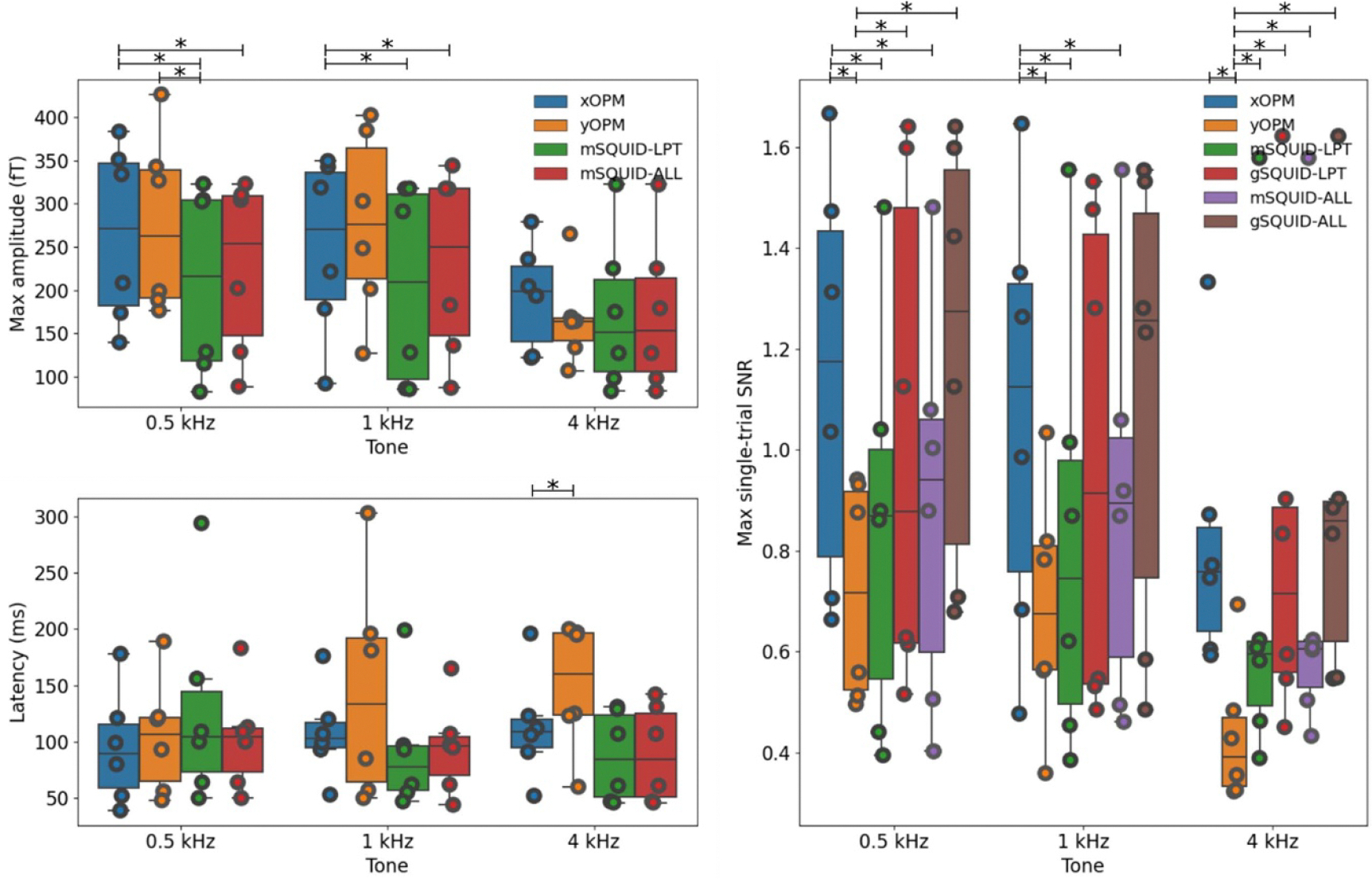
Boxplots comparing the maximum evoked-response amplitude and its latency as well as the estimated maximum single-trial SNR across the participants for different stimuli and sensor arrays. The maximum values are calculated across the sensors. Dots show the values for individual participants. For amplitude and latency, only magnetometers are compared. *p < 0.05 (Two-sided Wilcoxon signed-rank test).

Figure 3 summarizes the maximum response amplitudes and their latencies across the participants. The median maximum amplitude across the participants is larger for xOPM and yOPM than for mSQUID-LPT and mSQUID-ALL for all three tones. The result is statistically significant between xOPM and mSQUID-ALL for 0.5 and 1 kHz tones (Wilcoxon signed rank; two-sided; stat-value: 0; *p*-value: 0.03125). Figure 3 also compares the maximum estimated single-trial SNR over the sensor array across the participants. The figure shows that the maximum SNR is larger for xOPM than for yOPM for all tones (Wilcoxon signed rank; two-sided; stat-value: 0; *p*-value: 0.03125). The maximum SNR is higher for xOPM than for mSQUID-ALL for all three tones (statistically significant for 0.5 and 1 kHz tones; Wilcoxon signed rank; two-sided; stat-value: 0; *p*-value: 0.03125). gSQUID-ALL and gSQUID-LPT have similar or higher maximum SNRs than xOPM. For 0.5 and 4 kHz tones, gSQUID arrays have significantly higher maximum SNR than yOPM (Wilcoxon signed rank; two-sided; stat-value: 0; *p*-value: 0.03125).

### 3.2. Pairwise classification with LDA

**Figure 4.**
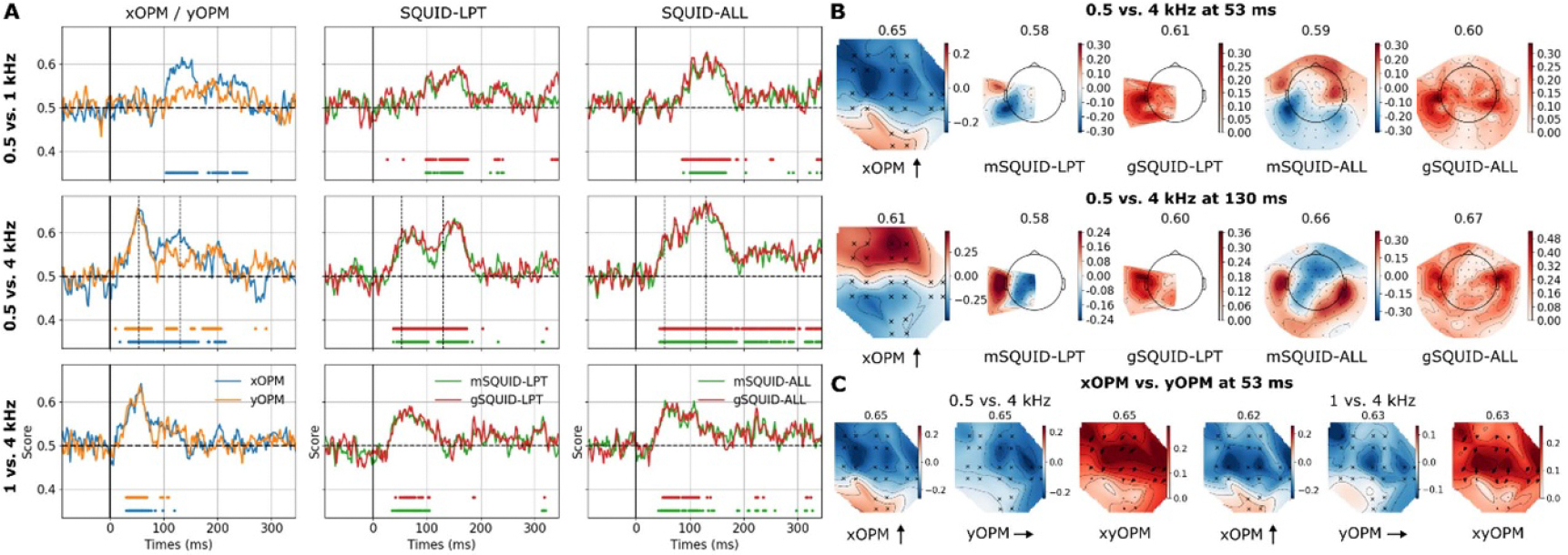
Pairwise classification of the single-trial evoked responses to auditory tones using linear discriminant analysis. Data for a single participant is shown (see figure 2). (A): Classification accuracy as a function of time. The points underneath the plots mark the time instances when the classification performance was significantly (*p* < 0.05; permutation test; FRD-correction *q* = 0.05) above the chance level (50%; dashed horizontal line). (B): Example field maps of the discriminant sources. Field maps are shown for two different tone pairs and time instances. The value on top of the plot shows the classification accuracy at that time instant. The time instances shown in panel B are indicated in panel A as dashed vertical lines. Positive/negative values are shown in red/blue, respectively. (C): The discriminant field patterns resolved by xOPM and yOPM at 53 ms and their combined fieldmap (xyOPM).

Figure 4(A) shows the results of the LDA-based pairwise classification of the tones as a function of time for a single participant (see figure 2). For this participant, all the sensor arrays except yOPM in 0.5 vs. 1 kHz classification achieve classification accuracy significantly higher than the chance level (50%; permutation test; FDR-correction *q* = 0.05; *p* < 0.05). The performance between SQUID magnetometers and gradiometers is similar. gSQUID-ALL/mSQUID-ALL perform better than gSQUID-LPT/mSQUID-LPT. The maximum classification accuracies across the sensor arrays are comparable with values ranging from 59% to 67%. For this participant, OPMs reach high classification accuracies early (M50) for 0.5 vs 4 kHz and 1 vs. 4 kHz.

Figures 4(B) and (C) shows examples of the LDA discriminant field patterns. For pairwise classification of 0.5 and 4 kHz tones at 53 ms (M50), OPMs yield higher classification accuracy than all the SQUID arrays. The mSQUID-LPT and gSQUID-LPT show a dipolar field pattern on the left hemisphere while mSQUID-ALL and gSQUID-ALL also show a dipole field on the right hemisphere. At this time instant, the inclusion of all the SQUID sensors and the right-hemisphere brain source does not considerably increase the classification accuracy (mSQUID-LPT: 58%; mSQUID-ALL: 59%). However, this is not the case at 130 ms, when the inclusion of the right-hemisphere source increases the classification accuracy by roughly 8% (mSQUID-LPT: 58%; mSQUID-ALL: 66%).

**Figure 5.**
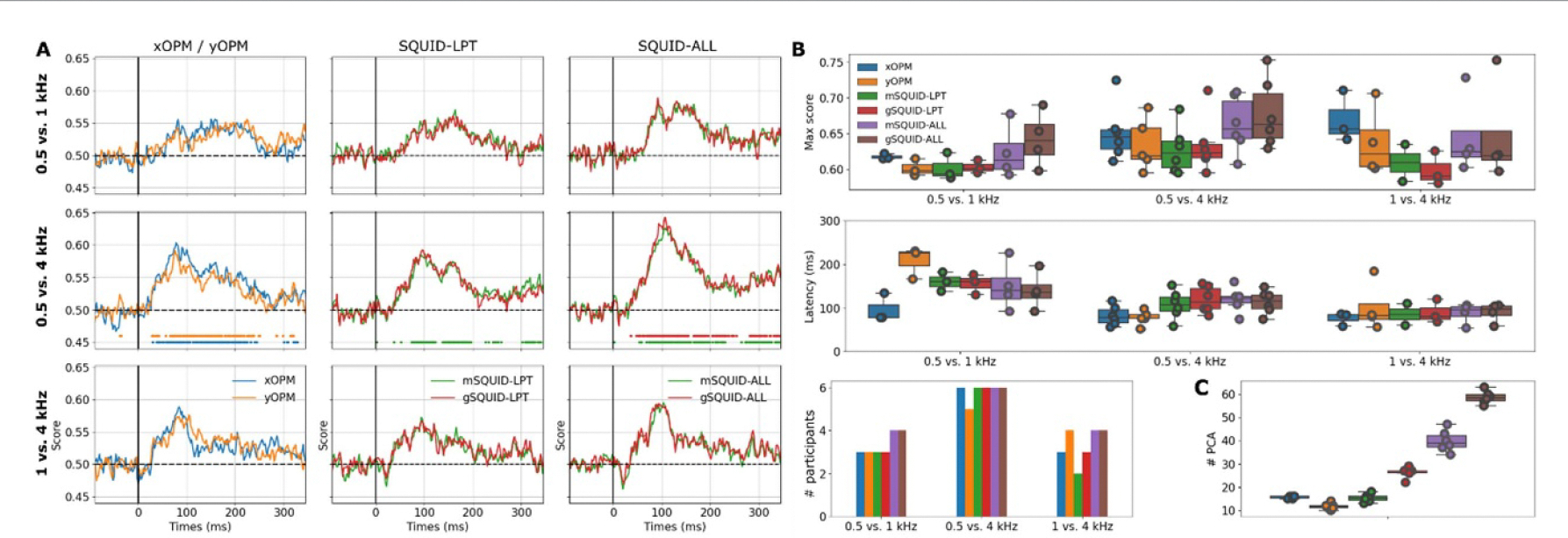
Pairwise classification of the single-trial evoked responses to auditory tones using linear discriminant analysis. The results are shown across the six participants. (A): The average classification accuracy across the participants as a function of time. The points underneath the plots show the time instances when the classification performance was significantly (*p* < 0.05; single-sided Wilcoxon signed-rank test; FDR-correction *q* = 0.05) above the chance level (50%; dashed horizontal line). (B): Summary of the classifier performance across the participants. The maximum significant (*p* < 0.05; permutation test; FRD-correction *q* = 0.05) classification accuracy and its latency are visualized using boxplots. The bar plot shows for each pairwise classification task the number of participants that showed a time instant with significant classification accuracy. (C): The number of principal (PCA) components needed to explain 99% of the data variance across the sensor arrays.

Figure 5(A) shows the LDA classification accuracy averaged over the participants as a function of time. Again, SQUID magnetometers and gradiometers have similar performance. The results are statistically significant (Wilcoxon signed rank; single-sided; FDR-correction *q* = 0.05; *p* < 0.05) only for classification between 0.5 and 4 kHz tones (all the arrays except gSQUID-LPT). The classification accuracy is comparable between the OPM arrays and the SQUID arrays covering the left-hand-side of the helmet (mSQUID-LPT/gSQUID-LPT): OPM arrays give slightly higher accuracy than those SQUID arrays for two of the classification tasks. The SQUID arrays covering the whole cortex give higher accuracy than the OPM arrays and mSQUID-LPT/gSQUID-LPT.

The statistically significant (permutation test; FDR-correction *q* = 0.05; *p* < 0.05) LDA classification results across the participants are summarized in figure 5(B). There are minor differences between the arrays in the number of participants that show significant classification accuracy; mSQUID-ALL and gSQUID-ALL yield the most participants with significant classification accuracy demonstrating the benefits of whole-head measurements. OPM provide significant accuracy in slightly more participants than mSQUID-LPT and gSQUID-LPT. Across the participants, OPMs give higher maximum classification accuracies than mSQUID-LPT and gSQUID-LPT (e.g. for 0.5 vs. 4 kHz xOPM: 65%, gSQUID-LPT: 62%; for 1 vs. 4 kHz xOPM: 66%, gSQUID-LPT: 59%). In addition, xOPM yields higher maximum accuracies than yOPM. The poor noise performance of the yOPM is attributed to the lower shielding factor of the person-sized shield along its longitudinal axis (*y*-axis). SQUIDs covering the whole head (mSQUID-ALL/gSQUID-ALL) have higher accuracies than OPMs for classification of two of the tone pairs.

Figure 5(C) shows boxplots of the number of PCA components that explain 99% variance of the data across the participants and the arrays. The median numbers of those PCA components across the participants are 16, 11.5, 15.5, 27, 39 and 58.5 for xOPM, yOPM, mSQUID-LPT, gSQUID-LPT, mSQUID-ALL and gSQUID-ALL, respectively.

### 3.3. Multiclass classification with EEGNet and xDAWN spatial filtering

**Figure 6.**
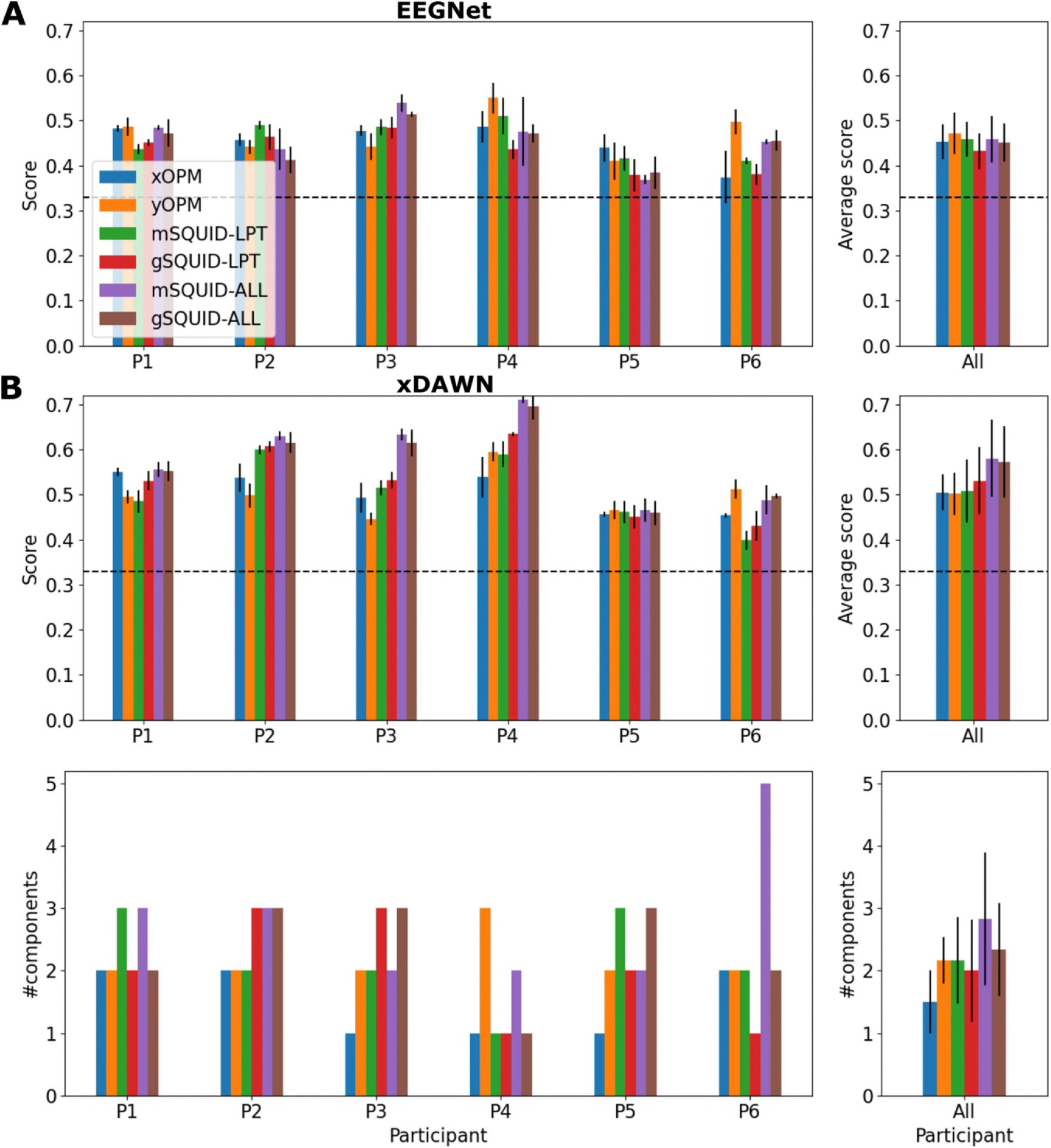
The performance of EEGNet convolutional neural network (A) and xDAWN spatial filtering with logistic regression (B) in multiclass classification of three auditory tones. In the left-hand plots the average accuracy over the four cross-validation folds is shown for each participant while in the right-hand- plots the average accuracy over the participants is visualized. The bottom panel in (B) shows the number of xDAWN spatial filters that gave the best accuracy for each participant. The horizontal dashed line indicates the classification chance level (33%).

The obtained multiclass classification accuracies with EEGNet and xDAWN spatial filtering are shown in figure 6. Across the six participants, all arrays show greater classification accuracy than the chance level (33%) with statistical significance (Wilcoxon signed rank; single-sided; stat-value: 21; *p*-value: 0.016) both with EEGNet and xDAWN methods. The average accuracy with EEGNet is 45.3%, 47.1%, 45.8%, 43.2%, 45.9% and 45.1% for xOPM, yOPM, mSQUID-LPT, gSQUID-LPT, mSQUID-ALL and gSQUID-ALL, respectively. The corresponding accuracies with xDAWN are 50.5%, 50.2%, 50.8%, 53.1%, 58.1% and 57.2%, respectively. We note that the accuracies given by EEGNet and xDAWN are not directly comparable due to different amount of training data. When comparing the classification accuracies across the arrays, the differences between xOPM-mSQUID-ALL and xOPM-gSQUID-ALL with xDAWN are statistically significant (Wilcoxon signed rank; two-sided; stat-value: 0; *p*-value: 0.03125). On average, two xDAWN spatial filters gave the best performance for all the arrays, except for xOPM and mSQUID-ALL for which 1–2 and 3 spatial filters gave the best performance, respectively.

## 4. Discussion

We compared the performance of OPM- and SQUID-MEG systems in classifying single-trial responses to auditory tones at three frequencies. For two of the tones, the tangential OPM sensors gave higher peak amplitudes than the normal-component-measuring SQUID magnetometers, as could be expected due to the closer proximity to the brain (however, not trivially true due to different measurement directions). The maximum estimated single trial SNR was higher in OPMs than in SQUID magnetometers; SNRs between OPMs and SQUID planar gradiometers were similar, demonstrating that gradiometric measurements of the normal field component provide a higher SNR than direct measurement of the field even after application of signal-space separation for interference rejection.

Temporal LDA classification analysis showed that OPMs provided higher classification accuracies than SQUIDs having a similar coverage of the left auditory cortex. However, when including all SQUID sensors in the helmet, the SQUID classification scores were larger than those for OPMs for two of the tone pairs. This demonstrates the benefit of a whole-head measurement of the neuromagnetic field: the sources in the right auditory cortex can positively influence the classification performance.

In multiclass classification with EEGNet and xDAWN spatial filtering, both OPMs and SQUIDs gave classification accuracies significantly higher than the chance level for all participants. The differences between OPM and SQUID arrays were small with EEGNet: OPMs gave slightly better performance than SQUIDs. With xDAWN decoding, SQUIDs covering the whole head performed the best with a higher margin than with EEGNet.

While SQUID planar gradiometers had higher maximum estimated single-trial SNRs than SQUID magnetometers, their classification performance was similar (see, e.g. figure 5). This indicates that at least partly the information recorded by them is dependent. One reason for this is their partly overlapping lead fields. In terms of amplitude, SNR and classification performance, the on-scalp tangential field components (xOPM vs. yOPM) showed more variability than SQUIDs with a suggestion that xOPM provided better performance than yOPM. The variability between xOPM vs. yOPM demonstrates that the two approximately orthogonal tangential field components couple differently to brain activity. Theoretically, both xOPM and yOPM are most sensitive to neural currents directly below the sensor but with orthogonal lead fields (e.g. Iivanainen et al 2017). Due to the alignment of the auditory cortex, the dipole sources are typically oriented towards or away from top of the head (e.g. Albrecht et al 2000) aligned with the sensitive axes of yOPM sensors. Dipole sources with such orientation would generate magnetic field along xOPM explaining partly the slightly better performance of xOPM over yOPM. Moreover, in our setup yOPM measures more external interference due to its alignment with the longitudinal axis of the cylindrical person-sized magnetic shield: the longitudinal axis of the shield has the lowest shielding factor reducing SNR of yOPM.

The variability between xOPM and yOPM demonstrates the benefit of measuring multiple components of the magnetic field. In future, it would be of interest to compare the classification performance of OPM arrays that measure one, two or three components of the field. Triaxial OPMs (Boto et al 2022) can measure all three orthogonal components of the field at the expense of a small loss in sensor sensitivity. It could be argued that triaxial sensors should provide the best performance both in classification and interference rejection (Brookes et al 2021) if their SNR is not severely degraded compared to a single-axis measurement.

The motivation of our study is similar to that by Andersen et al (2020): to use the array’s classification performance to quantify the spatio-temporal resolution between on-scalp (here OPM) and SQUID-MEG. Andersen et al (2020) showed that the classification performance of their on-scalp MEG device was better than that of SQUID-MEG for somatosensory stimulation for the P16m response (10–20 ms) while it was worse for the P60m response (50–70 ms). The authors reported that the observed P16m response in on-scalp MEG was surprising as it is not typically observed in (SQUID-)MEG. In contrast to their study, we did not necessarily observe any new signals with our OPM system but however showed that the OPMs can yield higher classification accuracies for responses seen both in OPMs and SQUIDs (see, e.g. the M50 response in figure 4).

Altogether, the results presented here demonstrate that the current (partial-coverage) OPM-MEG system can acquire comparable or slightly better data than a conventional SQUID-MEG system processed with state-of-the-art interference suppression methods. OPMs are still a maturing technology and new developments are expected on many fronts ranging from sensor improvements (sensitivity, triaxial field sensing; Brookes et al 2021, Boto et al 2022), array construction (optimized spatial sampling; Beltrachini et al 2021, Iivanainen et al 2021, Yeo et al 2022, Zhdanov et al 2023) and signal and data processing. Such developments will pave the way for high bandwidth non-invasive brain–computer interfaces.

## 5. Conclusions

We compared an OPM-MEG system to a commercial SQUID system in classifying participants’ single-trial evoked responses to three auditory tones. In pairwise temporal classification of the responses, OPMs had better performance than SQUIDs having similar coverage of the participant’s left hemisphere. SQUIDs covering the whole head had generally the best performance, demonstrating the benefits of whole-head measurements. The results suggest that the current OPM-MEG system yields high-quality brain data with potential for further improvements.

## Supplementary Material

Supp Material

Supplementary material for this article is available online

## Data Availability

The datasets generated during and/or analyzed during the current study are available from the corresponding author on reasonable request.
