# Supplementary material for: Single-trial classification of evoked responses to auditory tones using OPM- and SQUID-MEG
Source: J Neural Eng. Author manuscript; Available in PMC 2026 Jul 6. (PMC13333261; doi:10.1088/1741-2552/acfcd9)
Supplement: Supp Material [file NIHMS2185328-supplement-Supp_Material.pdf]

Supplementary material for Single-trial classification of evoked responses to auditory tones using OPM- and SQUID-MEG

Joonas Iivanainen[1*], Tony R. Carter[1], Michael C. S. Trumbo[1], Jim McKay[2], Samu Taulu[3], Jun Wang[4,5], Julia M. Stephen[6], Peter D. D. Schwindt[1] and Amir Borna[1]

[1]Sandia National Laboratories, PO Box 5800, Albuquerque, NM 87185–1082, United States of America

[2]Candoo Systems Inc., Port Coquitlam, BC, Canada

[3]University of Washington Seattle, Seattle, WA, United States of America

[4]Department of Speech, Language, and Hearing Sciences, The University of Texas at Austin, Austin, TX, United States of America

[5]Department of Neurology, The University of Texas at Austin, Austin, TX, United States of America

[6]The Mind Research Network a Division of Lovelace Biomedical Research Institute, Albuquerque, NM 87106, United States of America

[*]Correspondence: jaiivan@sandia.gov

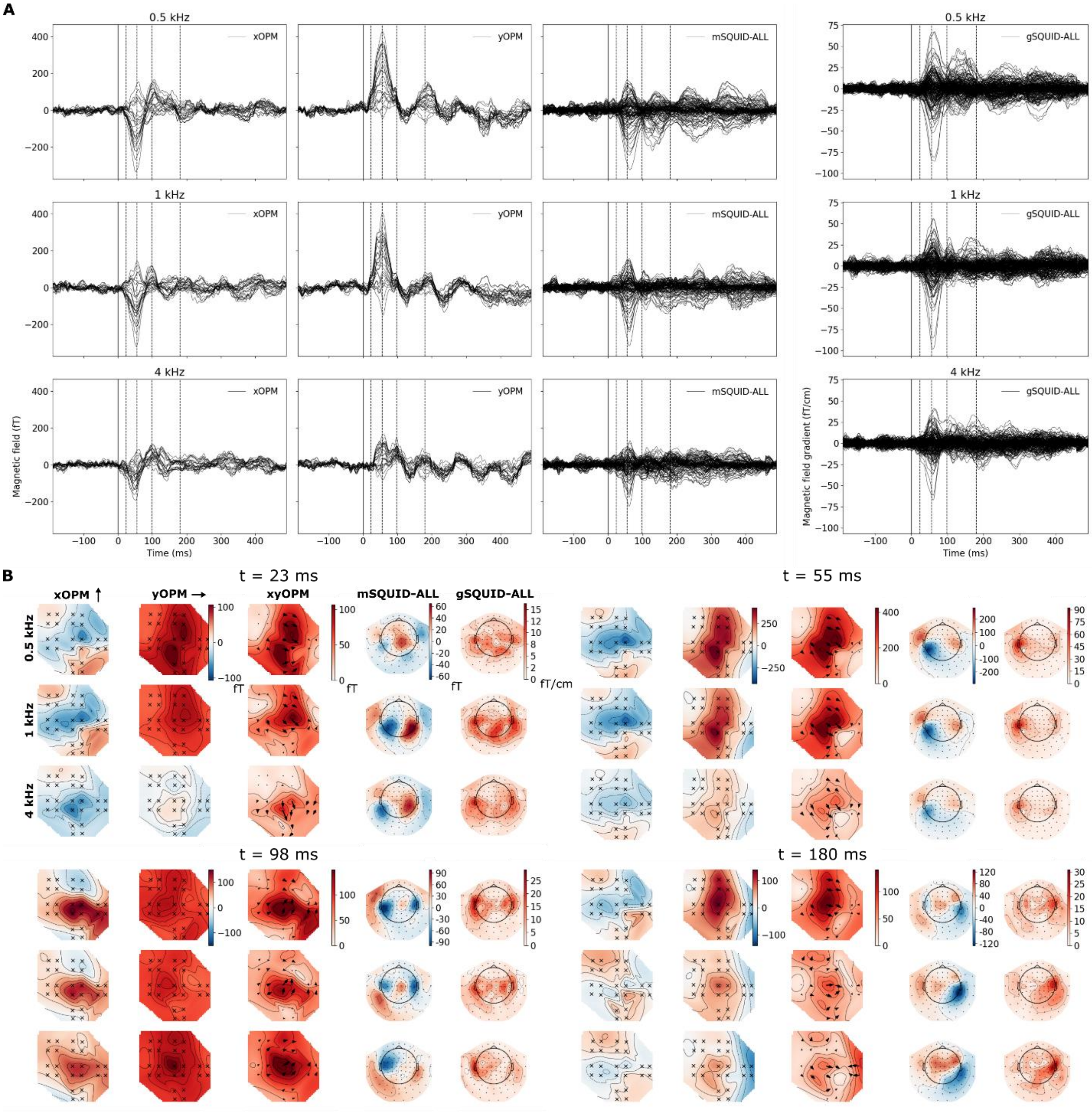

Figure 1: Single participant averaged evoked responses to 0.5, 1 and 4 kHz tones from participant 1 (P1). Please see Figure 2 in the main text for more information.

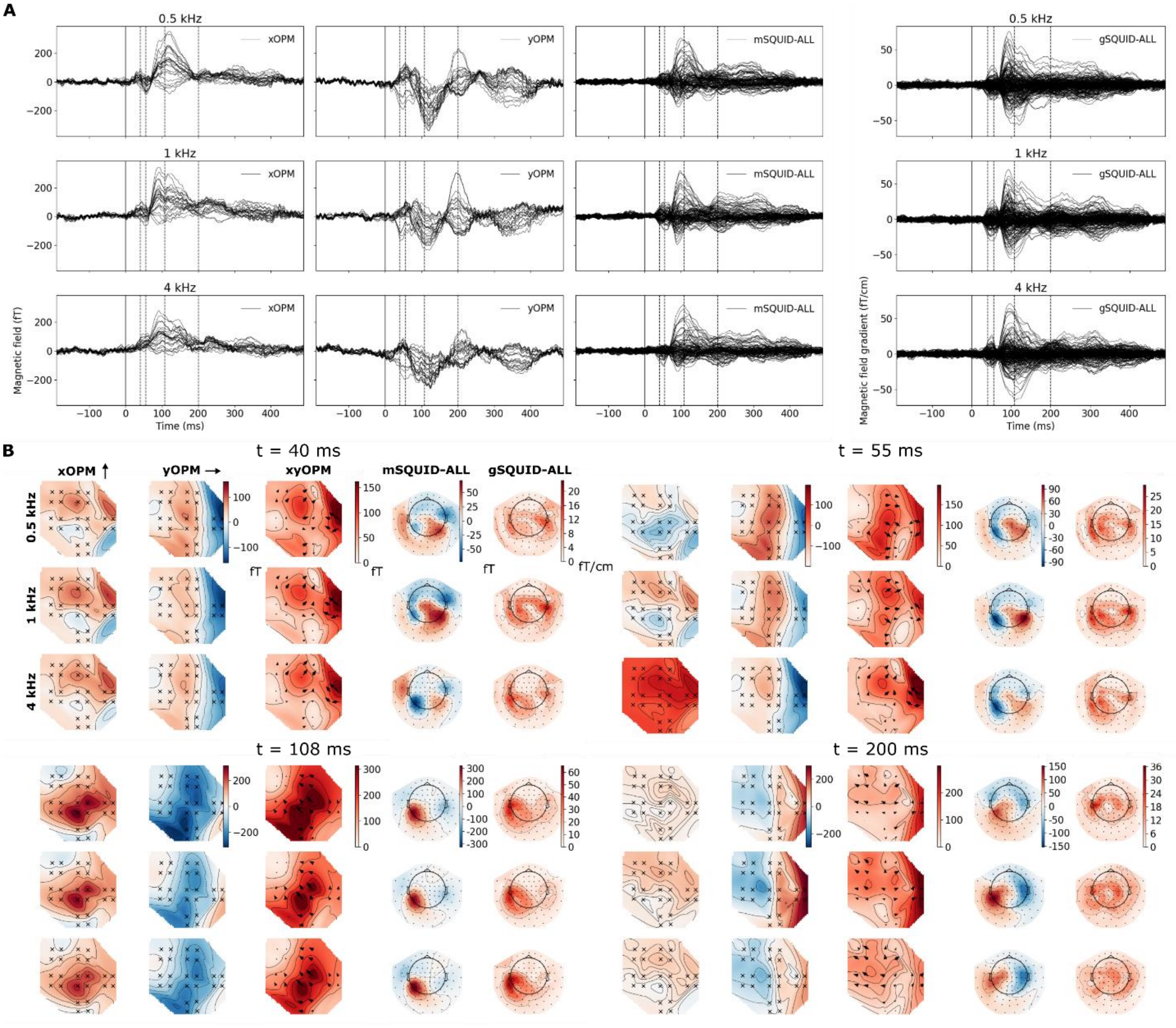

Figure 2: Single participant averaged evoked responses to 0.5, 1 and 4 kHz tones from P2. Please see Figure 2 in the main text for more information.

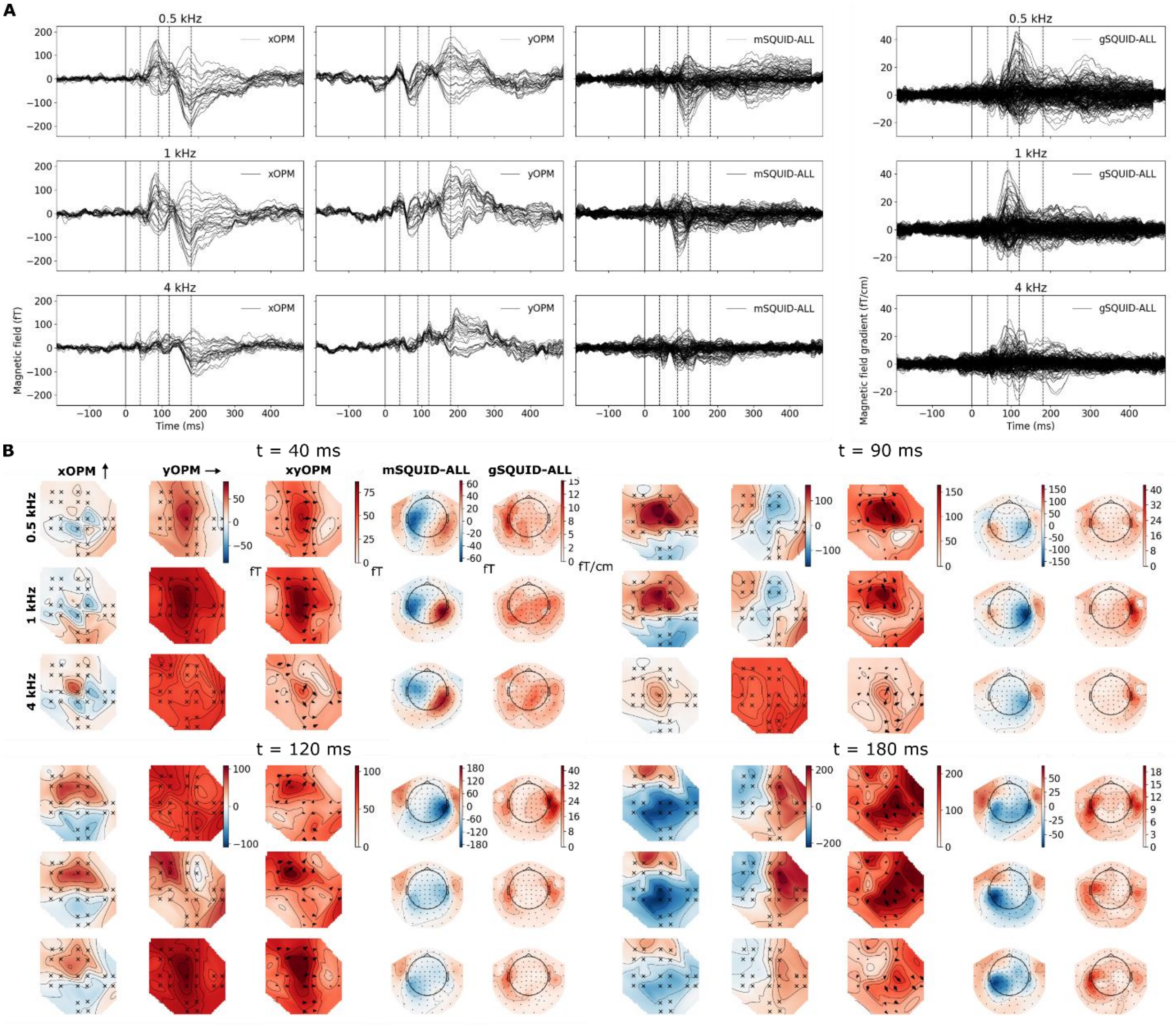

Figure 3: Single participant averaged evoked responses to 0.5, 1 and 4 kHz tones from P3. Please see Figure 2 in the main text for more information.

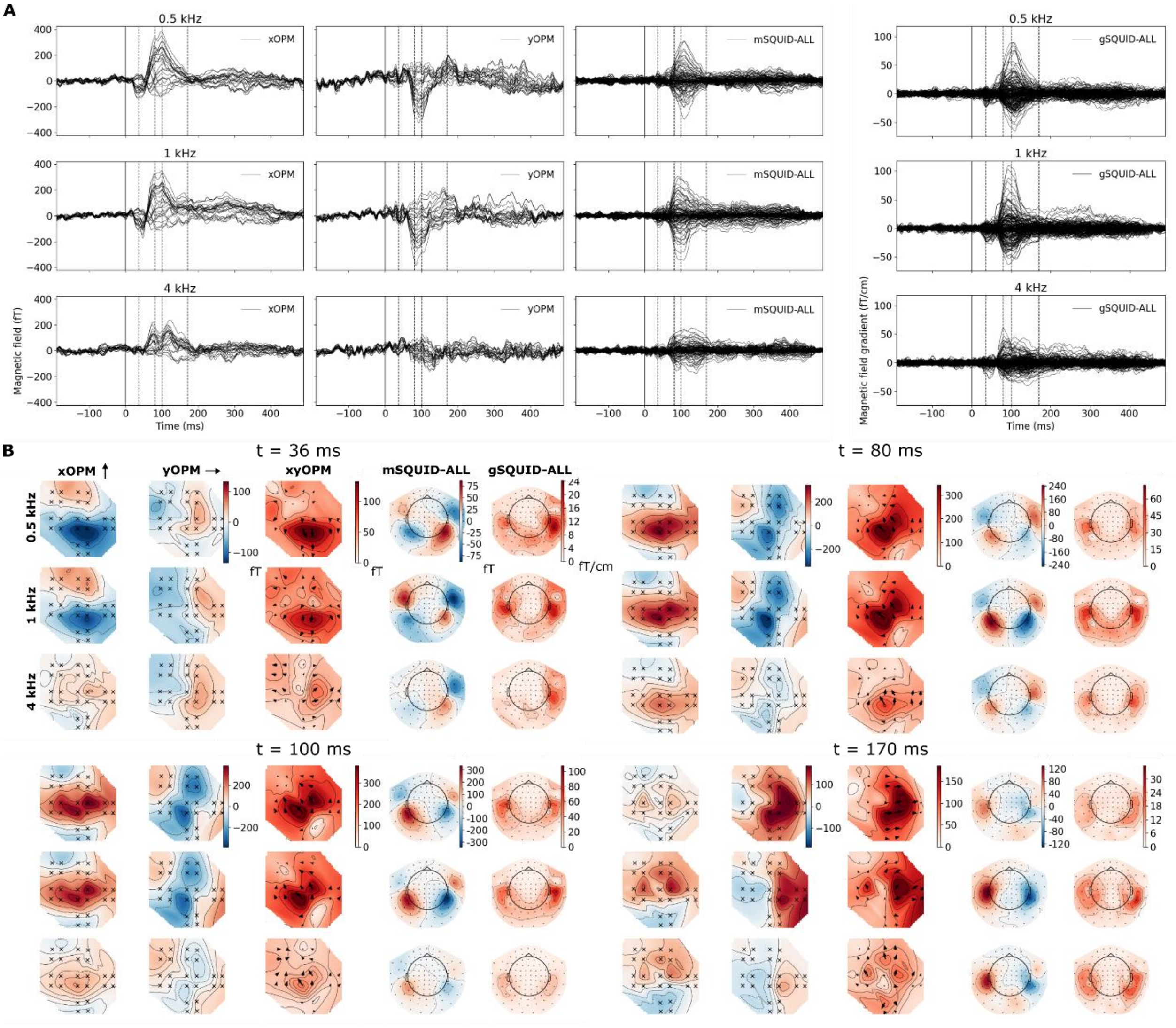

Figure 4: Single participant averaged evoked responses to 0.5, 1 and 4 kHz tones from P4. Please see Figure 2 in the main text for more information.

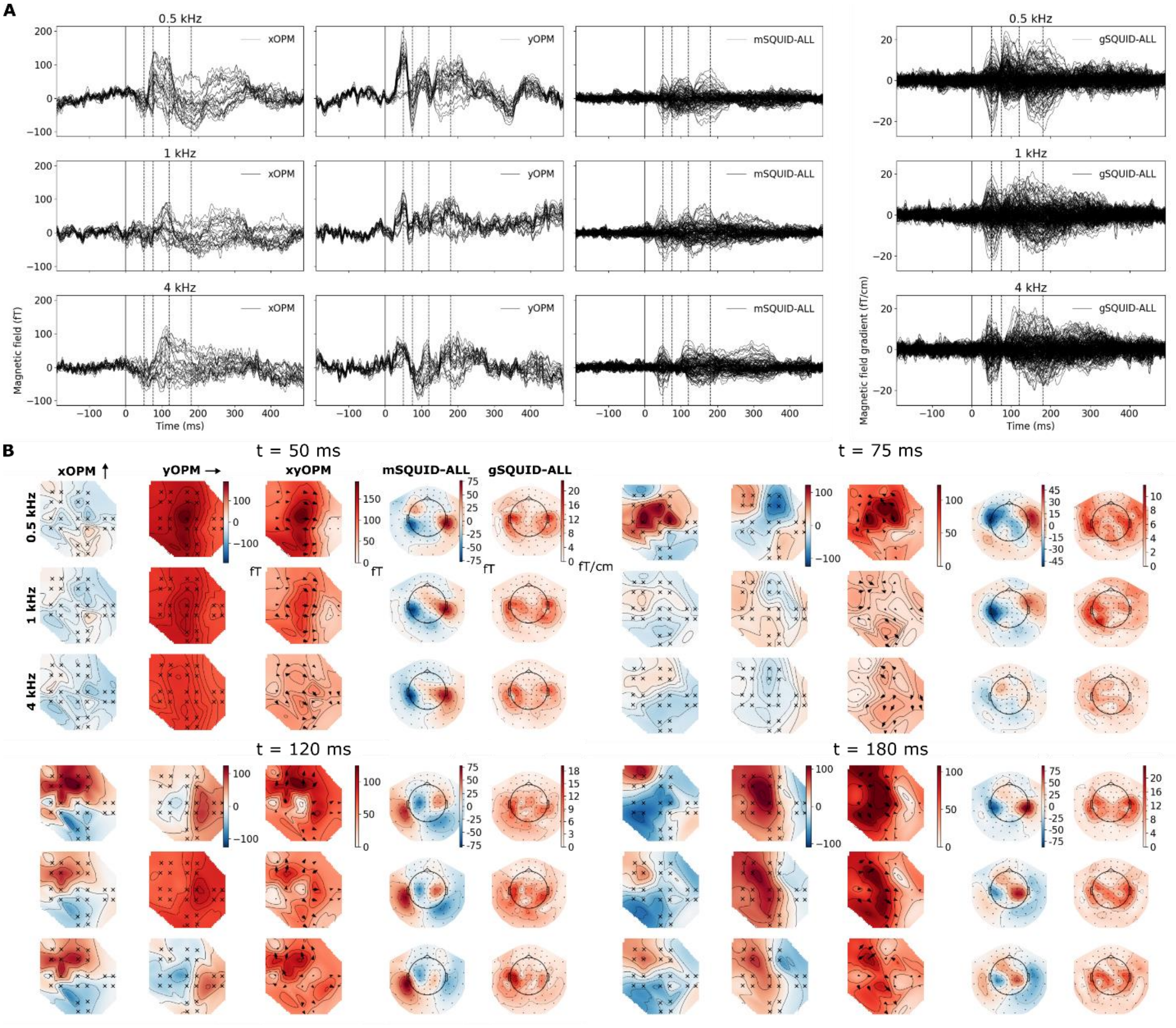

Figure 5: Single participant averaged evoked responses to 0.5, 1 and 4 kHz tones from P5. Please see Figure 2 in the main text for more information.

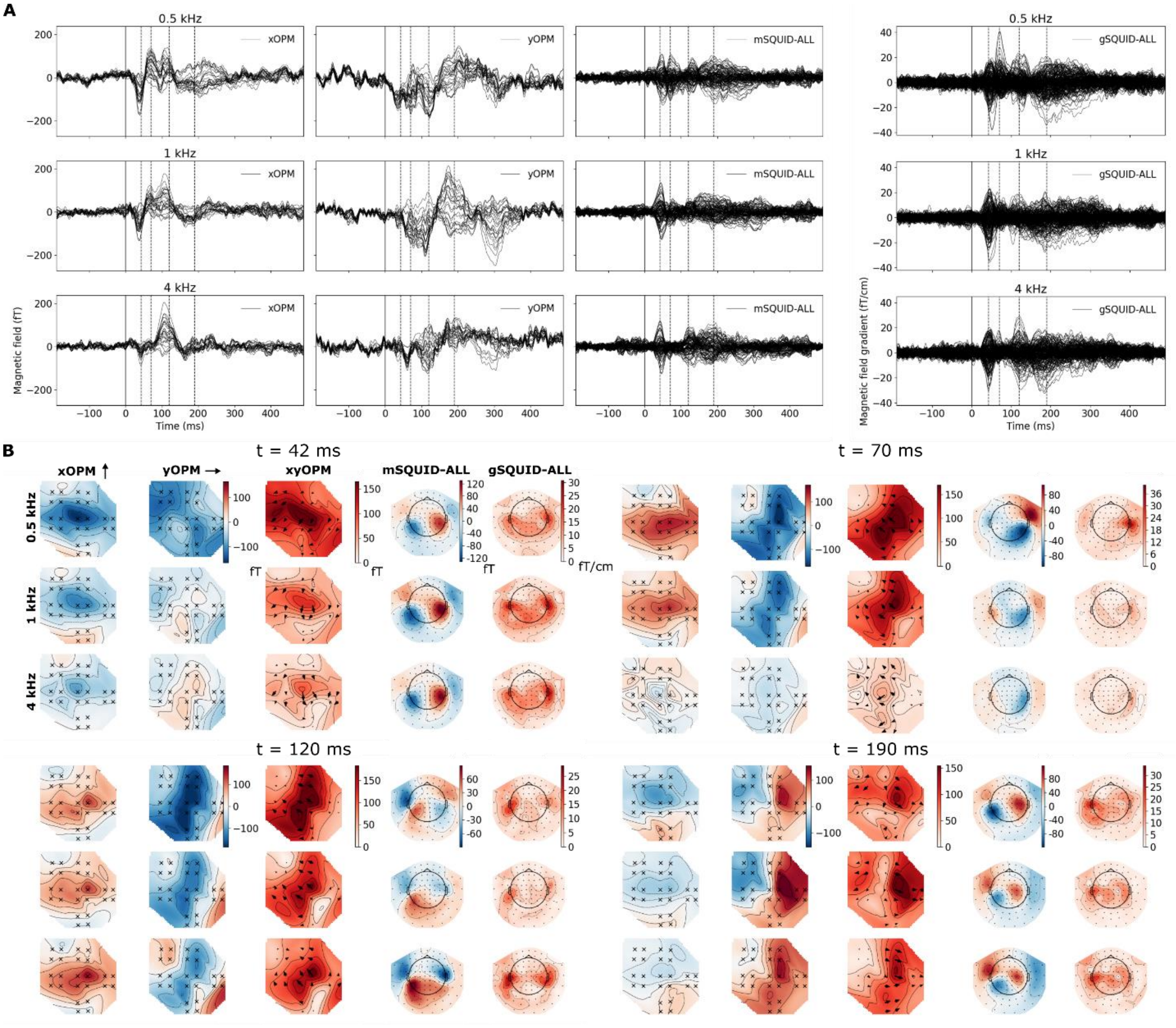

Figure 6: Single participant averaged evoked responses to 0.5, 1 and 4 kHz tones from P6. Please see Figure 2 in the main text for more information.

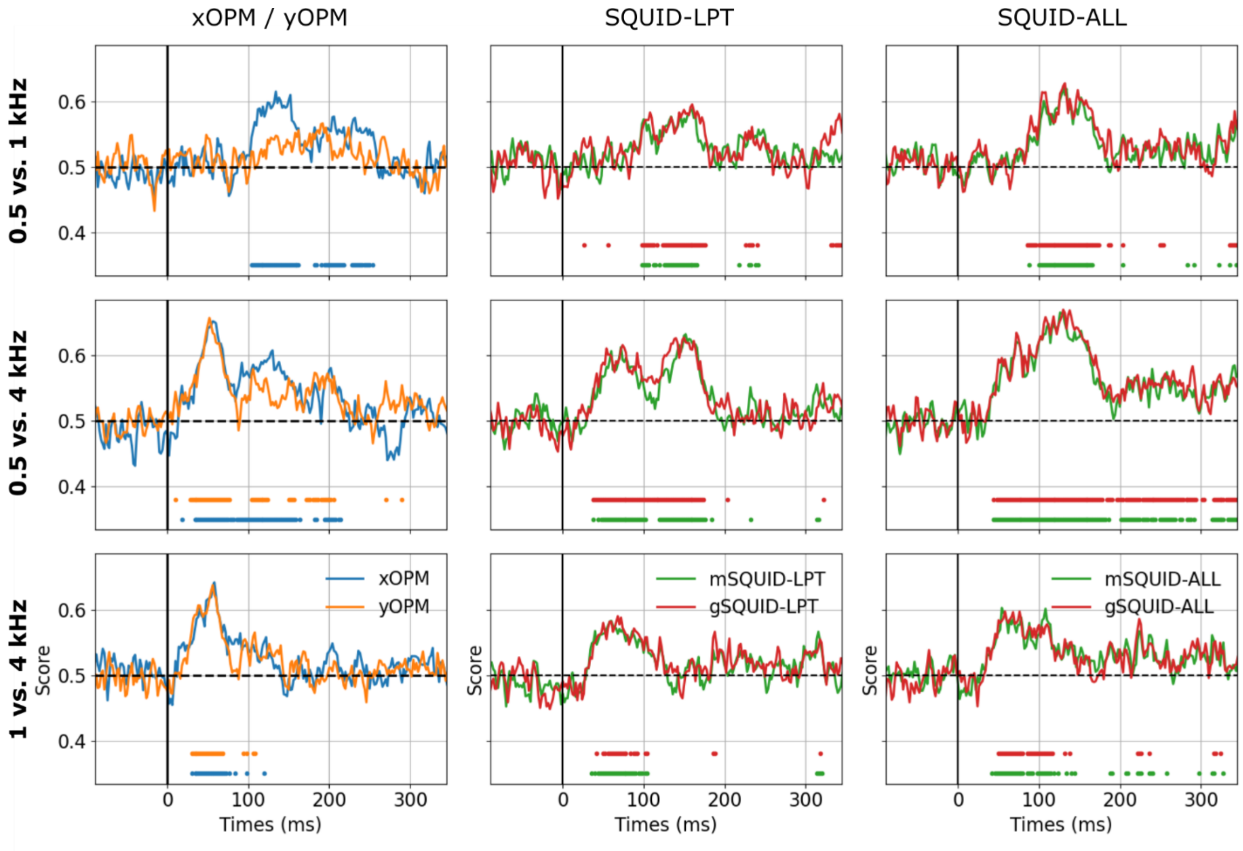

Figure 7: Pairwise classification of the single-trial evoked responses to auditory tones using linear discriminant analysis for P1. Please see Figure 5 in the main text for more information.

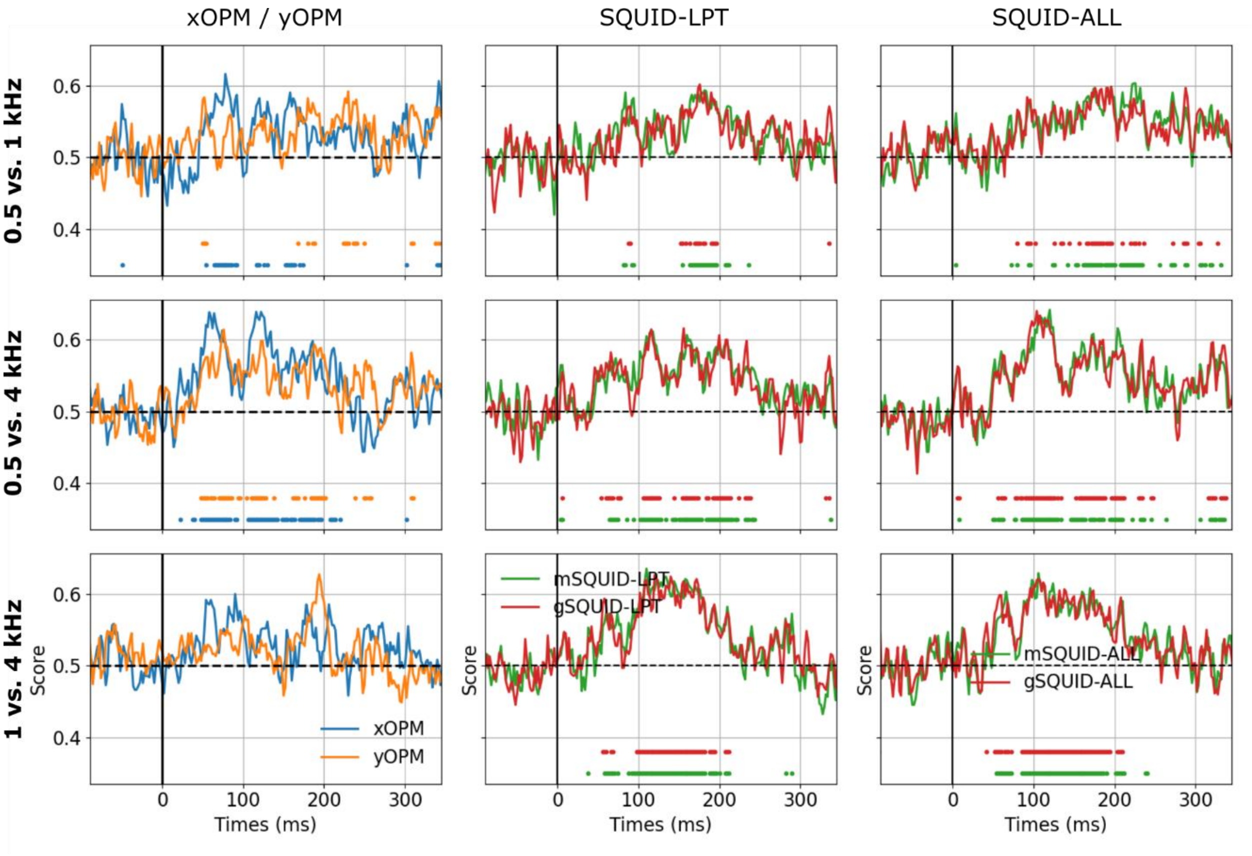

Figure 8: Pairwise classification of the single-trial evoked responses to auditory tones using linear discriminant analysis for P2. Please see Figure 5 in the main text for more information.

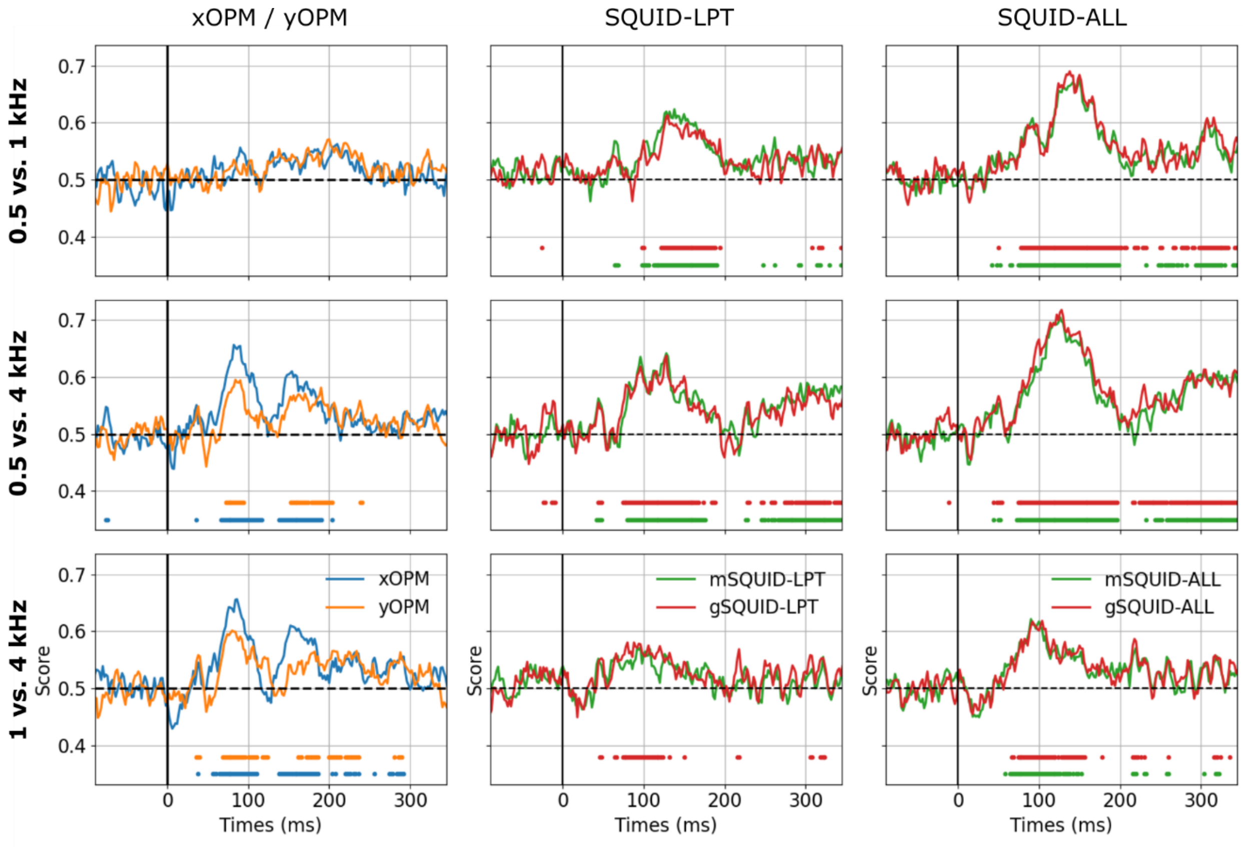

Figure 9: Pairwise classification of the single-trial evoked responses to auditory tones using linear discriminant analysis for P3. Please see Figure 5 in the main text for more information.

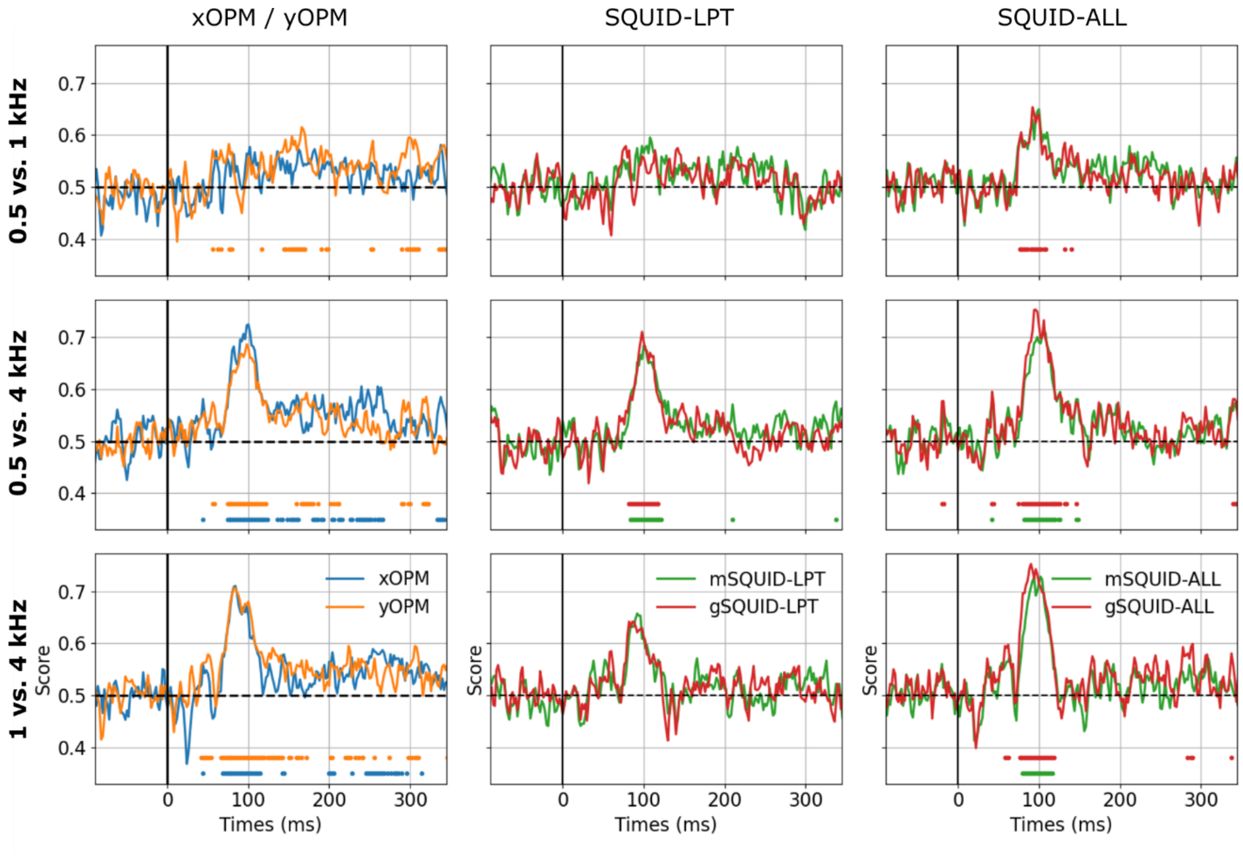

Figure 10: Pairwise classification of the single-trial evoked responses to auditory tones using linear discriminant analysis for P4. Please see Figure 5 in the main text for more information.

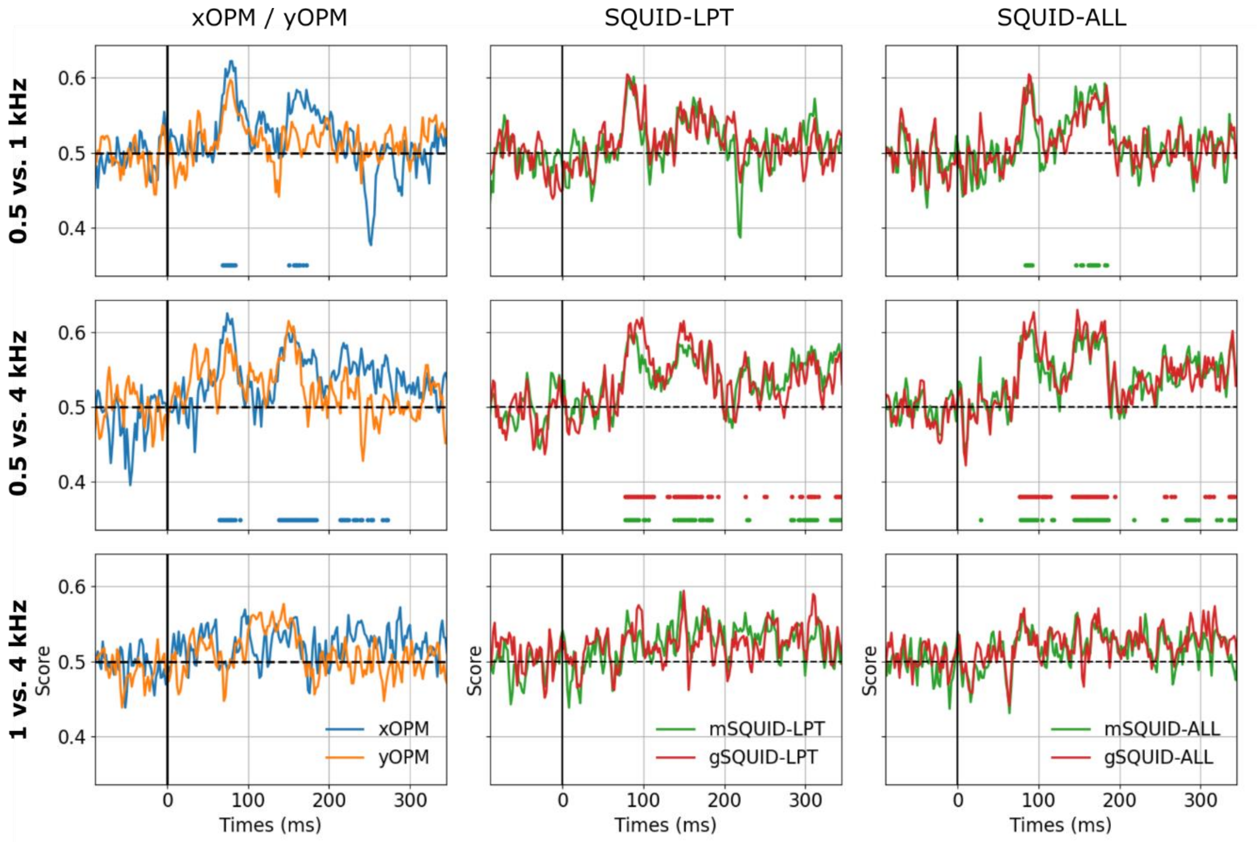

Figure 11: Pairwise classification of the single-trial evoked responses to auditory tones using linear discriminant analysis for P5. Please see Figure 5 in the main text for more information.

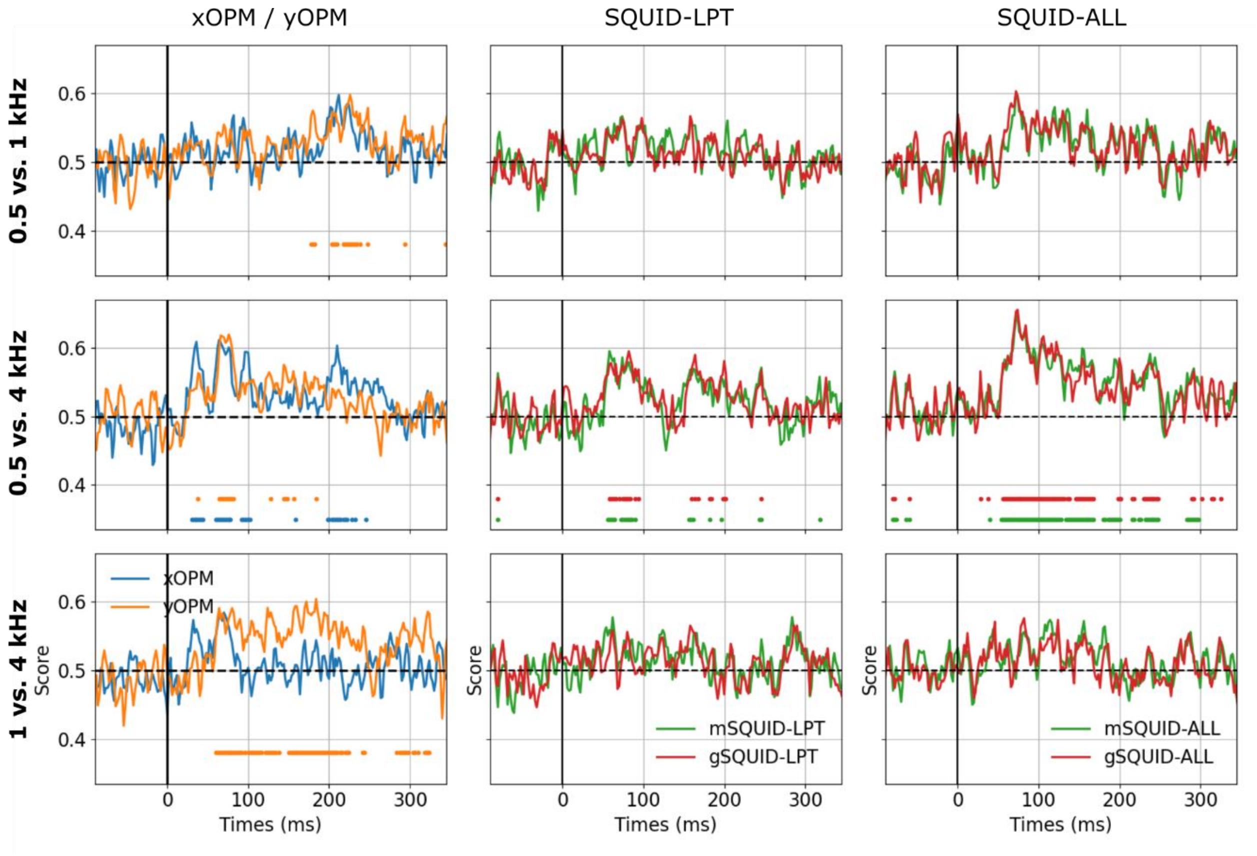

Figure 12: Pairwise classification of the single-trial evoked responses to auditory tones using linear discriminant analysis for P6. Please see Figure 5 in the main text for more information.
